# Constructing gene association networks for rheumatoid arthritis using the backward genotype-trait association (BGTA) algorithm

**DOI:** 10.1186/1753-6561-1-s1-s13

**Published:** 2007-12-18

**Authors:** Yuejing Ding, Lei Cong, Iuliana Ionita-Laza, Shaw-Hwa Lo, Tian Zheng

**Affiliations:** 1 Department of Statistics, Columbia University, New York, New York 10027, USA; 2 Department of Biostatistics, Harvard School of Public Health, Boston, Massachusetts 02115, USA

## Abstract

**Background:**

Rheumatoid arthritis (RA, MIM 180300) is a common and complex inflammatory disorder. The North American Rheumatoid Arthritis Consortium (NARAC) data, as part of the Genetic Analysis Workshop 15 data, consists of both genome scan and candidate gene studies on RA patients.

**Results:**

We applied the backward genotype-trait association (BGTA) algorithm to capture marginal and gene × gene interaction effects of multiple susceptibility loci on RA disease status. A two-stage screening approach was used for the genome scan, whereas a comprehensive study of all possible subsets was conducted for the candidate genes. For the genome scan, we constructed an association network among 39 genetic loci that demonstrated strong signals, 19 of which have been reported in the RA literature. For the candidate genes, we found strong signals for *PTPN22 *and *SUMO4*. Based on significant association evidence, we built an association network among the loci of *PTPN22*, *PADI4*, *DLG5*, *SLC22A4*, *SUMO4*, and *CARD15*. To control for false positives, we used permutation tests to constrain the family-wise type I error rate to 1%.

**Conclusion:**

Using the BGTA algorithm, we identified genetic loci and candidate genes that were associated with RA susceptibility and association networks among them. For the first time, we report possible interactions between single-nucleotide polymorphisms/genes, which may be useful for biological interpretation.

## Background

Rheumatoid arthritis (RA) is a heterogeneous disease with a complex genetic component. Previous studies identified multiple genetic regions that might be associated with RA. Amos et al. [1] identified strong linkage evidence on the major histocompatibility complex (MHC) region, 2q33 (*CTLA4*) and 11p12 in a genome scan. Plenge et al. [2] selected 14 genes that may be associated with RA or related autoimmune disorders and carried out a case-control study on these candidate genes, with significant results on *PTPN22*, *CTLA4*, and *PADI4*.

A common approach used in most association studies is to search, in a marker-by-marker fashion, for loci in association with the disease. This approach precludes consideration of interactions between genetic loci, resulting in loss of information that is important in understanding complex traits. On the other hand, consideration of high-dimensional interactions makes the computational complexity unrealistically high for large-scale studies. To address these difficulties, Lo and Zheng [3,4] showed the backward haplotype transmission association (BHTA) method for case-parent trios to be powerful for studying complex human disorders because it efficiently uses multilocus information. The method was extended in the backward genotype-trait association (BGTA) algorithm for case-control designs by evaluating association information on unphased multilocus genotypes [5]. In this paper, we applied BGTA to the Illumina genome scan (studied by Amos et al. [1]) and the candidate gene data (studied by Plenge et al. [2]) provided by NARAC as part of Problem 2 of Genetic Analysis Workshop 15.

## Methods

### Data processing

The Illumina data consist of 5407 single-nucleotide polymorphisms (SNPs) genotyped from 642 Caucasian families containing 1371 affected siblings with RA [1]. To apply the BGTA method, we first designated all unaffected people as our control group (*n *= 349). For families that did not contribute a control, we selected one case (*n *= 474). The level of missing values for the SNPs was <5%. The candidate gene data consist of 839 cases and 855 unrelated controls [2,6]. Genotypes on 20 SNPs in 14 candidate genes were included (Table 1). In Plenge et al. [2], the candidate gene data were checked for errors (~0.5%) and were found to be in Hardy-Weinberg equilibrium. There were 17 SNPs with less than 3% missing data. rs2240340 (*PADI4*) had ~65% missing (higher in the controls), which could potentially affect the results, and rs1061622 and 5509_5511delCAA had ~15% missing (also higher in the controls). For both data sets, we imputed the missing genotypes using fastPhase [6].

**Table 1:** 20 SNPs genotyped on the loci of 14 putative RA candidate genes

SNP	Gene	Locus
rs2476601	*PTPN22*	1p13.3–13.1
CT60	*CTLA4*	2q33
rs1061622	*TNFRSF1B*	1p36.3–36.2
rs2240340	*PADI4*	1p36.13
rs6149307	*HAVCR1*	5q33.2
5509_5511delCAA	*HAVCR1*	5q33.2
IGR2096ms1	*IBD5*	5q31.1
IGR3084ms1	*IBD5*	5q31.1
IGR3138ms1	*IBD5*	5q31.1
rs2073838	*SLC22A4*	5q31.1
rs31480	*IL3*	5q31.1
rs2243250	*IL4*	5q31.1
rs237025	*SUMO4*	6q25
rs577001	*SUMO4*	6q25
rs1248696	*DLG5*	10q23
HugotSNP12ms3	*CARD15*	16q21
HugotSNP8ms2	*CARD15*	16q21
HugotSNP13ms2	*CARD15*	16q21
rs2268277	*RUNX1*	21q22.3
rs755622	*MIF*	22q11.23

### BGTA screening

Given *k *SNP markers, there are 3^*k *^possible unphased genotypes. The association information score – the genotype-trait distortion (GTD) statistic – used by BGTA is defined on the sum of the squared difference between individual genotype's sample relative frequency among the cases and the controls, i.e.,

$$\text{GTD}={\left(1/n_{d}+1/n_{u}\right)}^{-2}{\sum_{i=1}^{3^{k}}\left(n_{i}^{d}/n_{d}-n_{i}^{u}/n_{u}\right)}^{2},$$

where *n*_*d *_and *n*_*u *_are total numbers of cases and controls, and $n_{i}^{d}$ and $n_{i}^{u}$ are counts of genotype *i *(on the *k *markers under study) among cases and controls, respectively. With the constant (1/*n*_*d *_+ 1/*n*_*u*_)^-2^, GTD has expectation 1 asymptotically under the null hypothesis of no association. If a marker is removed from the studied set, the GTD score might decrease or increase, thereby reflecting the contribution of that marker. The genotype-trait association (GTA) score for marker *M *given a current set of markers is defined as $\text{GTA(}M\text{)}=\frac{1}{2}\Delta \text{GTD}+\tilde{A}$, where ΔGTD is the GTD score without *M *minus the GTD with *M*, and $\tilde{A}$ is an adjusting term defined in [5] that makes GTA have expectation 0 when none of the markers in the subset is associated with the trait. When *M *is not associated with the disease but some of the selected markers are, GTA is positive, indicating an information gain that occurs when *M *(i.e., noise) is removed. If *M *is associated with the trait, GTA will be negative, indicating an information loss, and the magnitude of its value reflects the importance of *M*.

**Figure 1:**
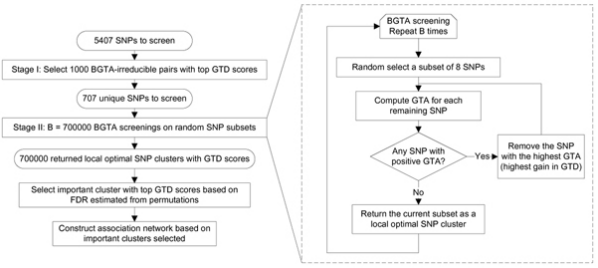
Flowchart for the analysis of the genome scan data.

Based on GTA, BGTA is a backward greedy search algorithm that removes markers that lead to information gain until no further gain is possible (see the flowchart in Figure 1). BGTA screening returns a small "optimal" cluster of markers with the peak GTD score. Herein, a subset is deemed *BGTA-irreducible *if no marker can be removed without lowering the GTD score. For a large number of markers, such a backward screening is not informative initially due to sparseness issues in high dimensions. Thus, BGTA has been implemented to screen a large number of random marker subsets [5]. In this paper, GTD scores of retained local optimal clusters are recorded, which measure the information content of each retained local optimal cluster. Local optimal clusters of SNPs with GTD score higher than a selection threshold are selected as important.

### Two-stage SNP selection

To overcome the computational complexity of analyzing 5407 SNPs while also considering interactions, we developed a two-stage SNP selection process (see Figure 1 for a flowchart). We assume that SNPs with high-dimensional interaction information will show some signal in pairwise GTD scores (this is an assumption that reduces computational burden). In Stage 1, we selected 1000 BGTA-irreducible pairs of SNPs with top GTD scores, which also included 22 SNPs with top marginal GTDs. This yielded 707 unique SNPs for the second stage, where we performed a regular BGTA screening on 700,000 random subsets of 8 SNPs. For discussion on the size of the subsets and the number of repeats, see Zheng et al. [5].

### Candidate gene study

For the candidate gene set, we evaluated a total of 2^20 ^- 1 GTD scores on all possible subsets of 20 SNPs (except for the empty set) to enumerate GTD's distribution for each subset size. Then we performed 30,000 greedy BGTA screenings on subsets of 8 SNPs to identify local optimal BGTA-irreducible SNP clusters.

### Selection threshold and evaluation of significance

**Figure 2:**
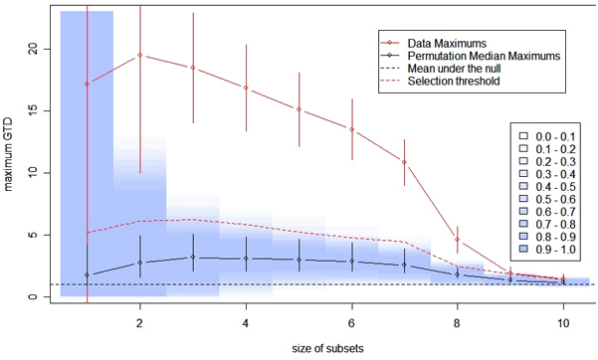
**Evaluation of significance of in the RA candidategene study**. For subsets of a given size, we plotted the highest GTD score (red solid line) with a 95% confidence interval (with Bonferroni correction for 2^20 ^- 1 multiple comparisons). The top GTD scores for sets of two to eight marker sets were significantly higher than the expected value under the null hypothesis (black dotted line at 1). Based on 100 permuted data sets, at different subset sizes, the black solid line displays the median maximum GTD scores and the vertical bars are the 95% confidence interval of permutations. For each permutation, we also calculated Bonferroni-corrected 95% confidence intervals for the maximum GTD scores. The blue shading indicates the coverage of these 100 confidence intervals at each subset size (the darkest being 0.9 to 1 (or 90 to 100%) and the lightest being 0.0 to 0.1 (or 0 to 10%)).

To estimate the distribution of GTD scores of local optimal BGTA-irreducible SNP clusters under the null hypothesis that no SNP is associated with the trait, we permuted the labels of disease status to create a simulated data set. For the two-stage study, we examined the GTD score density of returned clusters in the second stage from complete two-stage analyses of 50 permuted data sets. False discovery rate (FDR) was controlled by comparing the observed real density and the density under the null hypothesis (see [7-9]). For the candidate gene study, we set the selection threshold for each SNP subset size to be the maximum among 100 permuted replicates (the red dotted line in Figure 2) and identified local optimal BGTA-irreducible SNP subsets as significant at the 1% family-wise level. The relatively small number of permutations was due to the high computational burden of the analytical approach. This would result in non-trivial margin of error for the significance levels evaluated, which needs to be taken into consideration when interpreting the results.

### Association network construction

**Figure 3:**
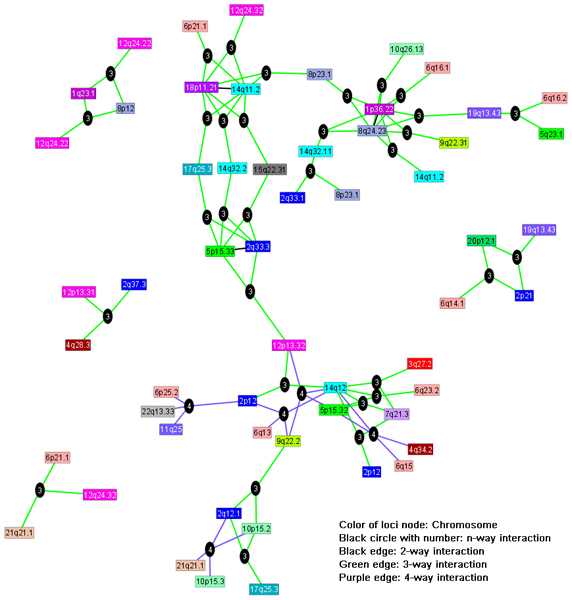
**Genome-wide association network for rheumatoid arthritis**. A different color represents each chromosome.

**Figure 4:**
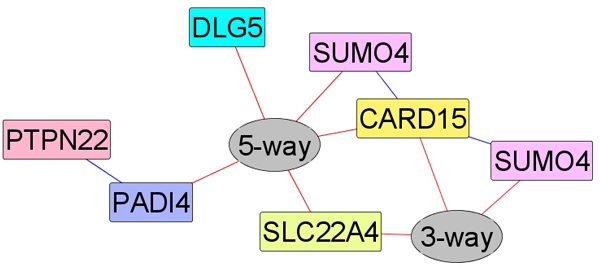
Association network of candidate gene loci with significant signals.

For subsets identified with more than one SNP, we constructed a graphical network using the graph exploration system GUESS [10] (Figures 3 and 4). A direct edge indicates a two-SNP cluster. For clusters with more than two SNPs, a non-SNP node was created with all involved SNP connected to it.

## Results and discussion

### Genome scan results

From the two-stage screening of the Illumina data, clusters of SNPs with high GTD scores were returned as important. Based on 50 permutations, we estimated the FDR as described by Storey and Tibshirani [7] at different GTD levels. Due to the small number of permutations, the estimated FDR has a high level of uncertainty for small FDR values. Therefore, we set the selection threshold of GTD to control for FDR at 30% for the association network construction while indicating in Table 2 SNPs with stronger signals at FDR = 15%. Using the individual clusters, we built an association network (Figure 3) based on the joint return clusters of those loci. One central and four satellite subnetworks are notable, and a number of loci seem to serve as hubs (e.g., 1p36.22 (*PADI4*), 2q33.3 (*CTLA4*), 5p15.33, 8q24.23, 14q11.2, 14q12, and 18p11.21), which have a large number of interaction edges with other loci. The literature suggests that seven out of these eight loci have previously been found to be associated with susceptibility to RA. All 39 marker loci in the association network of Figure 3 are listed in Table 2, about half of which were previously reported in the RA literature. One important RA susceptibility gene, *PTPN22*, was not among these 39 loci, but its signal was right below our selection threshold.

**Table 2:** Loci with a high joint GTD score identified in the genome scan

Locus	Reference
1p36.22 (*PADI4*)^a^	[1,11,12,2]
1q23.1	
2p21^a^	
2p12	[1]
2q12.1^a^	[12]
2q33.1–33.3 (*CTLA4*)^a^	[1,2]
2q37.3	
3q27.2	[14]
4q28.3	
4q34.2	
5p15.33–15.32^a^	[1]
5q23.1^b^	
6p25.2	
6p21 (*HLA-DRB1*)^a^	OMIM
6q13	[1,11]
6q14.1^a^	[1,11]
6q15	[1,11]
6q16.1–16.2^a^	[1,11]
6q23.2	
7q21.3	
8p23.1^a^	[13]
8p12	
8q24.23^a^	
9q22.2–22.31^a^	
10p15.3–15.2^a^	
10q26.13	
11q25	
12p13.32–13.31^a^	
12q24.22–24.32	
14q11.2^a^	[13]
14q12^a^	[13]
14q32.11–32.2^a^	
15q22.31^a^	[15]
17q25.3^a^	
18p11.21^a^	[16,12]
19q13.43	[16]
20p12.1^a^	[12]
21q21.1 (*RUNX1*)^a^	OMIM
22q13.33	

^a^Multiple SNPs in LD were found in significant clusters.

^b^This locus is 10 Mb away from *SLC22A4*.

### Candidate genes results

For the candidate gene data, we studied all possible subsets of SNPs and identified eight significant BGTA-irreducible subsets on seven SNPs after controlling for family-wise type I error. The marginally significant (one-marker subset) SNPs are rs2476601 (*PTPN2*2), and rs237025 and rs577001 (both at the locus of *SUMO4*). The significantly associated subsets with two or more SNPs represent possible interactions between these candidate genes in affecting the risk of developing RA. In Figure 4, red edges indicate interactions involving more than two SNPs, whereas blue edges indicate pairwise interactions between two SNPs. As previously identified in [2], we found PTPN22 and PADI4. However, the SNP at locus PADI4 has a high level of missing values, so results at this locus should be interpreted with caution.

Tables 3 and 4 illustrate a three-way interaction among *SLC22A4*, *SUMO4*, and *CARD15 *identified in our results. Table 3 lists the actual genotype distribution on these three SNPs (percentages out of 839 cases and 855 controls that have observed values). Table 4 displays the deviance table from the logistic regression between the disease status and these three SNPs including all possible interactions. Deviance can be directly used as a likelihood-ratio chi-squared test statistic that evaluates the contribution of each additional model term (main effect or interaction) [17]. From the logistic regression, the three-way interaction identified by BGTA is significant even when the main effects and two-way interaction terms were already included in the model. More interestingly, only one SNP at the *SUMO4 *locus demonstrated a significant main effect, indicating that contributions from *SLC22A4 *and *CARD15 *are mostly through interactions.

**Table 3:** Genotype distributions of identified SNPs on *SLC22A4*, *SUMO4*, and *CARD15 *among cases and controls^a^

	Percentage among cases or controls	
	
Genotype	Cases	Controls
1/1, 2/2, 2/3	0	0
1/1, 2/2, 3/3	0	1
1/1, 2/4, 2/3	0	0
1/1, 2/4, 3/3	0	0
1/1, 4/4, 2/3	0	0
1/1, 4/4, 3/3	0	0
1/3, 2/2, 2/3	0	1
1/3, 2/2, 3/3	6	6
1/3, 2/4, 2/3	0	0
1/3, 2/4, 3/3	6	7
1/3, 4/4, 2/3	0	0
1/3, 4/4, 3/3	3	2
3/3, 2/2, 2/3	1	1
3/3, 2/2, 3/3	28	36
3/3, 2/4, 2/3	1	1
3/3, 2/4, 3/3	**44**	**34**
3/3, 4/4, 2/3	0	0
3/3, 4/4, 3/3	9	11

^a^SNPs identified: rs2073838 (*SLC22A4*), rs577001 (*SUMO4*), HugotSNP12ms3 (*CARD15*)

**Table 4:** Logistic regression deviance table and likelihood ratio test results on data shown in Table 3

Model^a^	df	Deviance	Residual df	Residual deviance	*p*-Value from LR test
*Null model*			1670	2316.23	
*SLC22A4*	2	0.24	1668	2315.99	0.89
*SUMO4*	2	14.60	1666	2301.39	**0.0007^b^**
*CARD15*	1	0.08	1665	2301.31	0.78
*SLC22A4 *× *SUMO4*	4	10.40	1661	2290.92	**0.03**
S*LC22A4 *× *CARD15*	1	0.32	1660	2290.60	0.57
*SUMO4 *× *CARD15*	2	1.92	1658	2288.68	0.38
*SLC22A4 *× *SUMO4 *× *CARD15*	2	17.13	1656	2271.54	**0.0002**

^a^SNPs analyzed: rs2073838 (*SLC22A4*), rs577001 (*SUMO4*), HugotSNP12ms3 (*CARD15*)

^b^Bold indicates significant *p*-values.

Combining the results from the genome scan and the candidate genes results, *PTPN22 *had the strongest evidence as an RA susceptibility gene. *SLC22A4 *also showed up in the results of both studies.

## Conclusion

In this paper, the BGTA approach was applied to identify important genetic loci and gene × gene interactions on susceptibility to RA. Different analytical strategies were tailored for these two data sets of different nature, illustrating the applicability of BGTA and the GTD statistic to different studies. Using the BGTA method, both marginal and gene × gene interaction information were extracted and reflected in the GTD scores. Under a general analytical framework, both analyses result in association networks constructed based on gene clusters with significant association to RA. To overcome the dimensionality problems of a genome scan, we imposed a two-stage scheme based on BGTA screenings. For a small number of candidate genes, we used GTD directly on subsets of genes to identify clusters that were significantly associated with RA disease status. We addressed the multiple comparisons issue using the most direct permutation-based evaluation and controlled the FDR and the family-wise type I error rate. Both association networks identified in this paper demonstrated evidence on gene × gene interaction in affecting the risk of developing RA. Visualization of these networks displays interesting structures that could be used to generate testable biological hypotheses.

## Competing interests

The author(s) declare that they have no competing interests.

## References

[B1] Amos CI, Chen WV, Lee A, Li W, Kern M, Lundsten R, Batliwalla F, Wener M, Remmers E, Kastner DA, Criswell LA, Seldin MF, Gregersen PK (2006). High-density SNP analysis of 642 Caucasian families with rheumatoid arthritis identifies two new linkage regions on 11p12 and 2q33. Genes Immun.

[B2] Plenge RM, Padyukov L, Remmers EF, Purcell S, Lee AT, Karlson EW, Wolfe F, Kastner DL, Alfredsson L, Altshuler D, Gregersen PK, Klareskog L, Rioux JD (2005). Replication of putative candidate-gene associations with rheumatoid arthritis in >4,000 samples from North America and Sweden: association of susceptibility with *PTPN22*, *CTLA4*, and *PADI4*. Am J Hum Genet.

[B3] Lo SH, Zheng T (2002). Backward haplotype transmission association (BHTA) algorithm – a fast multiple-marker screening method. Hum Hered.

[B4] Lo SH, Zheng T (2004). A demonstration and findings of a statistical approach through reanalysis of inflammatory bowel disease data. Proc Natl Acad Sci USA.

[B5] Zheng T, Wang H, Lo SH (2006). Backward genotype-trait association (BGTA)-based dissection of complex traits in case-control designs. Hum Hered.

[B6] Scheet P, Stephens M (2006). A fast and flexible statistical model for large-scale population genotype data: applications to inferring missing genotypes and haplotypic phase. Am J Hum Genet.

[B7] Storey JD, Tibshirani R (2003). Statistical significance for genomewide studies. Proc Natl Acad Sci USA.

[B8] Efron B (2004). Large-scale simultaneous hypothesis testing: the choice of a null hypothesis. J Am Stat Assoc.

[B9] Storey JD (2002). A direct approach to false discovery rates. J R Statist Soc B.

[B10] Adar E (2006). GUESS: a language and interface for graph exploration. CHI'06: Proceedings of the SIGCHI Conference on Human Factors in Computing Systems.

[B11] John S, Shephard N, Liu G, Zeggini E, Cao M, Chen W, Vasavda N, Mills T, Barton A, Hinks A, Eyre S, Jones KW, Ollier W, Silman A, Gibson N, Worthington J, Kennedy GC (2004). Whole-genome scan, in a complex disease, using 11,245 single-nucleotide polymorphisms: comparison with microsatellites. Am J Hum Genet.

[B12] Osorio YFJ, Bukulmez H, Petit-Teixeira E, Michou L, Pierlot C, Cailleau-Moindrault S, Lemaire I, Lasbleiz S, Alibert O, Quillet P, Bardin T, Prum B, Olson JM, Cornélis F (2004). Dense genome-wide linkage analysis of rheumatoid arthritis, including covariates. Arthritis Rheum.

[B13] MacKay K, Eyre S, Myerscough A, Milicic A, Barton A, Laval S, Barrett J, Lee D, White S, John S, Brown MA, Bell J, Silman A, Ollier W, Wordsworth P, Worthington J (2002). Whole-genome linkage analysis of rheumatoid arthritis susceptibility loci in 252 affected sibling pairs in the United Kingdom. Arthritis Rheum.

[B14] Fisher SA, Lanchbury JS, Lewis CM (2003). Meta-analysis of four rheumatoid arthritis genome-wide linkage studies: confirmation of a susceptibility locus on chromosome 16. Arthritis Rheum.

[B15] Wise CA, Bennett LB, Pascual V, Gillum JD, Bowcock AM (2000). Localization of a gene for familial recurrent arthritis. Arthritis Rheum.

[B16] Kenealy SJ, Herrel LA, Bradford Y, Schnetz-Boutaud N, Oksenberg JR, Hauser SL, Barcellos LF, Schmidt S, Gregory SG, Pericak-Vance MA, Haines JL (2006). Examination of seven candidate regions for multiple sclerosis: strong evidence of linkage to chromosome 1q44. Genes Immun.

[B17] Agresti A (2002). Categorical Data Analysis.

